# Towards developing a test of global motion for use with Paralympic athletes

**DOI:** 10.1038/s41598-020-65202-x

**Published:** 2020-05-21

**Authors:** James W. Roberts, Benjamin Thompson, Susan J. Leat, Kristine Dalton

**Affiliations:** 1 University of Waterloo, School of Optometry & Vision Science, 200 University Avenue West, Waterloo, N2L 3G1 Ontario, Canada; 2 Liverpool John Moores University, Brain & Behaviour Laboratory, Research Institute of Sport & Exercise Sciences (RISES), Byrom Street, Tom Reilly Building, L3 5AF Liverpool, United Kingdom

**Keywords:** Health services, Quality of life

## Abstract

The Paralympic classification system for visual impairment only assesses static visual acuity and static visual field despite many Paralympic sports being dynamic in nature. As a first step towards determining whether motion perception tests should be used in Paralympic classification, we assessed whether motion coherence thresholds could be measured when visual acuity or visual fields were impaired at levels consistent with the current Paralympic classification criteria. Visual acuity and visual field impairments corresponding to Paralympic classification criteria were simulated in normally sighted individuals and motion coherence thresholds were measured. Mild-to-moderate visual acuity impairments had no effect on motion coherence thresholds. The most severe Paralympic class of acuity impairment (≥2.6 logMAR) significantly elevated thresholds. A trend towards superior motion coherence thresholds in the peripheral visual field compared to the central visual field was also present. Global motion perception appears to be measurable under simulated visual impairments that are consistent with the Paralympic classification. Poorer global motion perception was found for visual acuities >2.6 logMAR and visual fields <10° in diameter. Further research is needed to investigate the relationship between global motion perception and sports performance in athletes with real visual impairment.

## Introduction

The current classification of vision impairments for all Paralympic sports (except Shooting Para Sport, which has recently established a new classification system)^1^ is based on static visual acuity and visual field loss in the better eye only. However, the most recent International Paralympic Committee Classification Code (2007, 2015), mandated that classification rules for Para sports should be sport-specific and evidence based, which means the classification systems for athletes with vision impairment need to be reviewed^2–4^. Expert consensus recently identified “establishing the most appropriate measures of vision impairment to be used for classification (e.g., contrast sensitivity, motion perception, or other sport-specific tests developed for classification) as a top priority^5^. Therefore, classification assessments may need to be expanded to measure a wider range of visual functions relevant to sport performance. Motion perception is involved in all dynamic sports and was identified as one of the vision impairment assessments that should be considered for use in classification. Currently, there is little known about how vision impairments affect motion perception.

Global motion perception relies on area MT/V5 (dorsal visual pathway) and involves the integration of local motion signals from V1 into a coherent motion percept^6–12^. Motion coherence thresholds are common measure of global motion perception and involve the presentation of random dot kinematograms (RDKs) that are constructed from two sets of moving dots. One set moves in a single coherent direction (signal dots), while the other set moves in a random direction (noise dots). Participants judge the direction of the signal dots, as the percentage of signal dots (signal to noise ratio) is varied. The signal to noise ratio required for threshold task performance is known as the motion coherence threshold.

Motion coherence thresholds measured using RDKs are relatively robust to changes in RDK element spatial frequency^13^, moderate reduction in acuity (≤0.7 logMAR) induced by optical blur^14^, and reduced supratheshold contrast^15,16^. In addition, motion coherence thresholds and other types of complex motion perception can be measured in the peripheral visual field^14,17^. This suggests that motion coherence thresholds could be measured in individuals with low vision due to visual acuity and/or visual field loss.

Preliminary pilot data collected from national level Para sport athletes with vision impairments also suggests that motion coherence thresholds can be measured in individuals with low vision (see Appendix A, Supplementary Material). While these pilot data appear promising, the sample size was small (n = 5), and the vision impairments of the athletes did not span the entire range of acceptable vision impairments for Paralympic competition (visual acuities of 1.0 to >2.6 logMAR; visual field radius <20 degrees).

The aim of this study was to assess whether motion coherence thresholds could be measured when visual acuity or visual fields were impaired to the levels required for Paralympic classification (Table 1), a much broader range of visual impairment than used in previous studies, to determine the feasibility of including motion coherence thresholds in future visual impairment classification research. To achieve this aim, we simulated visual acuity and visual field impairments in participants with normal vision. We then measured coherence thresholds for translational and radial motion using wide-field RDKs with large dots. It was hypothesized that motion coherence thresholds would be measureable with simulated low vision at the levels of impairment currently required for Paralympic classification.

**Table 1:** International Paralympic Committee (IPC) classifications for visual impairment^2^.

Classification	Visual Acuity	Visual Field
B1	≥2.6 logMAR	n/a
B2	≥1.5 to <2.6 logMAR	<10° diameter
B3	≥1.0 to <1.5 logMAR	<40° diameter

## Results

### Simulated acuity

**Figure 1:**
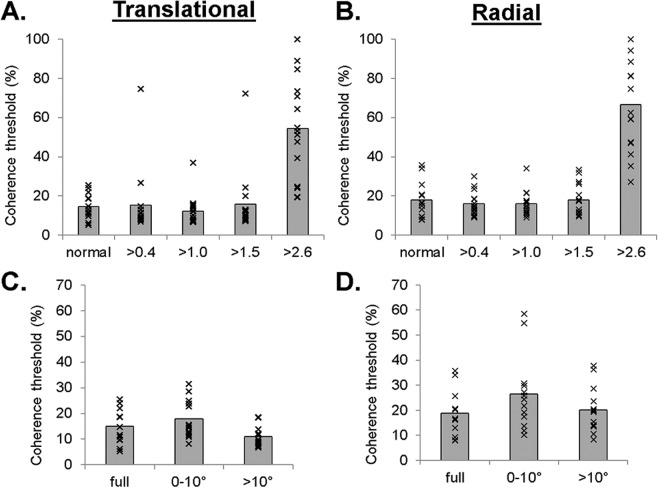
Mean motion coherence thresholds (%) with individual participant data (x) as a function of simulated visual acuity impairment (normal, >0.4, >1.0, >1.5, >2.6) and simulated visual field impairment (full, 0–10°, >10°). (**A**) Translational motion-simulated visual acuity; (**B**) radial motion-simulated visual acuity; (**C**) translational motion-simulated visual field; (**D**) radial motion-simulated visual field.

Table 2 presents the visual acuity thresholds obtained as a result of the simulated visual acuity losses. Initial evaluation of the single dot detection task with the ≥2.6 logMAR simulation revealed 100% accuracy (out of a total 8 responses) in all the participants tested. For coherence thresholds, ANOVA (2 motion × 5 acuity levels) revealed a significant main effect of motion type, *F*(1, 14) = 5.38, *p* < 0.05, partial *ƞ*^2^ = 0.28, indicating a lower threshold with translational motion (*M* = 22.49%, *SD* = 11.44) compared to radial motion (*M* = 26.88%, *SD* = 6.94). There was also a significant main effect of simulated acuity, *F*(4, 56) = 63.91, *p* < 0.01, partial *ƞ*^2^ = 0.82 (Fig. 1A,B), but there was no significant motion × acuity interaction, *F*(4, 56) = 1.29, *p* = 0.29, partial *ƞ*^2^ = 0.08. Post hoc analysis revealed a significant threshold increase in the ≥2.6 logMAR condition (*M* = 60.48%, *SD* = 21.00) compared to all other acuity conditions (combined *M* = 15.74, *SD* = 7.30). There were 7 participants (2 translational; 5 radial) who were unable to complete at least one global motion trial at 100% coherence during the ≥2.6 logMAR condition.

**Table 2:** Means, standard deviations, maximum and minimum logMAR visual acuities obtained for each target visual acuity threshold following the application of Bangerter foils and/or laminate sheets.

	normal	≥0.4	≥1.0	≥1.5	≥2.6
Mean	−0.18	0.55	1.16	1.57	2.72
SD	0.08	0.06	0.05	0.03	0.06
Max	0.02	0.64	1.20	1.62	2.86
Min	−0.28	0.46	1.08	1.52	2.66

For response time, ANOVA showed a significant main effect of motion type, *F*(1, 14) = 8.11, *p* < 0.05, partial *ƞ*^2^ = 0.37, indicating a shorter time to respond to the translational (*M* = 2.44 s, *SD* = 1.00) compared to radial motion (*M* = 2.93 s, *SD* = 1.23). There was a significant main effect of simulated acuity, *F*(4, 56) = 4.95, *p* < 0.05, partial *ƞ*^2^ = 0.26, but no significant motion × acuity interaction, *F*(4, 56) = 0.67, *p* > 0.62, partial *ƞ*^2^ = 0.05. Post hoc analysis did not reveal any significant differences in response time between acuity conditions (all *p* > 0.05).

### Simulated field

**Figure 2:**
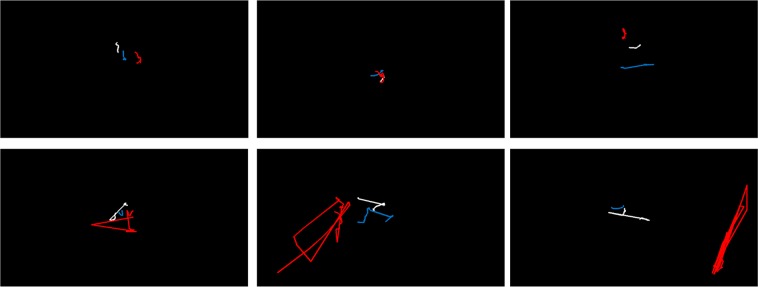
Example gaze position traces of representative individual participants at select trials (10–20^th^ response step in staircase) for translational motion stimuli. Full, 0–10° and >10° visual field conditions are represented by the *white*, *red*, and *blue lines*, respectively. *Top panel* illustrates cases of predominantly central fixation with minor search. *Bottom panel* illustrates cases of overt searches away from centre only during the 0–10° field condition. Note, images are scaled to actual display size of the experiment.

Two additional participants had to be removed from the simulated visual field impairments analysis due to a lost eye tracker signal in at least one condition (final n = 13) (Fig. 2 shows fixation patterns). ANOVA (2 motion × 3 field) for coherence threshold showed a significant main effect of motion type, *F*(1, 12) = 20.49, *p* < 0.001, partial *ƞ*^2^ = 0.63, which indicated a lower threshold for translational motion (*M* = 14.62%, *SD* = 4.86) compared to radial motion (*M* = 21.88%, *SD* = 6.76). The main effect of field approached significance, *F*(2, 24) = 3.16, *p* = 0.061, partial *ƞ*^2^ = 0.21, whereby full fields (*M* = 16.95%, *SD* = 6.58) and fields >10° (*M* = 15.55%, *SD* = 5.22) had numerically lower thresholds than the 0–10° field condition (*M* = 22.24%, *SD* = 10.54) (Fig. 1C,D). There was no significant motion x field interaction, *F*(2, 24) = 1.32, *p* = 0.29, partial *ƞ*^2^ = 0.10.

For response time, there was no significant main effect of motion type, *F*(1, 12) = 0.29, *p* = 0.60, partial *ƞ*^2^ = 0.02, or simulated field, *F*(2, 24) = 0.64, *p* = 0.54, partial *ƞ*^2^ = 0.05 and there was no significant motion x field interaction, *F*(2, 24) = 2.62, *p* = 0.09, partial *ƞ*^2^ = 0.18 (*M* = 2.41 s, *SD* = 1.11).

## Discussion

We examined the impact of simulated visual acuity and visual field impairments on global motion perception across a much broader range of visual impairment than previous studies. Simulated impairments were chosen based on the current Paralympic classification criteria for visual impairment, which utilise assessments of static visual acuity and visual field. In addition, we simulated visual field restrictions in the presence of free eye movements, and thus closely replicated real-life field losses. We found that motion coherence thresholds could be measured when visual acuity or visual fields were impaired to the levels required for Paralympic classification. We also observed some differences across the categories of visual impairment (B1-B3) whereby simulated visual acuity deficits >2.6 logMAR (B1) and/or central visual field deficits <10° (B2) elevated motion coherence thresholds. When considered in conjunction with the pilot data on national Para sport athletes (Appendix A, Supplementary Material) our results suggest that motion coherence thresholds could be considered for incorporation into Paralympic classification research.

Our findings are consistent with previous evidence demonstrating partial^18^ or complete^14^ ability to perceive global motion in the presence of a severe simulated visual impairment. Burton *et al*. (2015) identified some losses in global motion perception following simulated low visual acuity, although not to the same extent as global form perception^18^. On the other hand, Zwicker *et al*. (2006) revealed no systematic differences in motion coherence thresholds following the application of positive blurring lenses^14^, but these may not have decreased visual acuity to the same degree as the current study. Together, these results support previous observations that low spatial frequency information, that is less affected by blur than high spatial frequency information, is sufficient to support global motion perception^13,19^.

That being said, motion coherence thresholds greatly increased following the most severe simulated acuity impairment of >2.6 logMAR (20/7962), in which the simulated acuity exceeded the resolution (but not the detection) acuity of the dots (which subtended a single limb width of 1.7 logMAR). These findings suggest that global motion processing was limited by difficulty in differentiating individual dots within the RDK. This reasoning is consistent with the two-stage process of global motion perception^20^, whereby local motion is processed prior to motion integration. However, global motion perception was still measurable at the most severe visual acuity impairment with a mean motion coherence threshold of approximately 60%. This further supports the potential for global motion measurements as a useful measure for Paralympic athletes with low vision.

In regard to the influence of visual field, there was a trend towards increasing motion coherence thresholds during the central (10°) field condition compared to the full and peripheral (>10°) field conditions. This finding corroborates previous evidence of alterations to motion perception following field-related impairments^21^. What’s more, global motion perception has been shown to withstand effects of stimulus eccentricity providing local dot details are perceptible within the periphery^13^. It is precisely this feature of the visual field that may explain the gaze activity as the eyes appeared to scan more during peripheral field loss (see Fig. 2). Presumably, there was an attempt to compensate for this field restriction by capturing local dot details within the central field. However, due to the minor statistical effects and comparatively limited range of visual fields (0–10°, >10°), future investigation is recommended on this matter.

### Limitations of the study

There are differences between simulated and true visual impairment. Those with true visual impairment may have multiple deficits and there is likely wide variability between individuals due to the age of onset of the visual impairment and its cause (ocular, cortical), which may affect the development of visual processing. These multiple deficits cannot be simulated with a decrease of visual acuity or visual field. However, data from a small sample of individuals with true visual impairment (Appendix A, Supplementary Material) are consistent with the results of our simulation study. Further studies of participants with true visual impairment are required to assess the relationship between motion coherence thresholds and Para sport performance.

## Methods

### Participants

Eighteen participants took part in the study (mean age = 24.3 years ± SD 5.3, range 18–40 years). All participants had normal or corrected-to-normal vision (mean acuity = −0.18 logMAR ± SD 0.08, range = −0.28 to +0.02). All participants had had a full eye exam less than two years before the date of the first study visit and none of the participants had any ocular or neurological conditions (based on self-report). The study was designed in accordance with the Declaration of Helsinki and has been reviewed and received ethics approval through a University of Waterloo Research Ethics Committee. Informed consent was obtained from all participants prior to their participation in the study.

### Apparatus

Stimuli were generated via Matlab (The Mathworks Inc., Natick, MA) running Psychtoolbox on a Lenovo Thinkpad P50 with NVIDIA Quadro M2000M graphics (temporal resolution = 60 Hz, spatial resolution = 1920 ×1080 pixels), and displayed on a gamma-corrected 50” Sony Bravia 3D LED television (Model: KDL-50W800C). The stimulus aperture reached within 174.5 mm of the screen edge to subtend a visual angle of 44.58 × 26.81° at a 1 m viewing distance. The RDK featured 100 white dots (mean dot luminance = 119 cd/m^2^, dot density = 0.058 dot/deg^2^) on a black background moving at a velocity of 6°/s. There was a 5% chance of the dots disappearing upon each screen refresh (~16 ms). Each dot subtended a visual angle of 0.83° (14.54 mm), which equated to a single limb width of a 1.7 logMAR optotype for letter acuity. This size was chosen because pilot data indicated that a single dot of this size could be detected with a visual acuity ≥2.6 logMAR. It was possible to use dots that were smaller than the worst visual acuity impairment simulated because the visual acuity for detecting stimuli is better than the visual acuity for resolving stimuli details^22^.

RDKs were presented with either translational or radial motion and consisted of white dots on a black background. For translational motion, the dots moved vertically up or down to avoid any contamination by horizontal nystagmus in future studies involving participants with low vision^23^. For radial motion, a 1.4° region in the centre of the display remained blank^24^ and the dots moved inwards or outwards. Dots wrapped-around when they reached the edge of the stimulus aperture.

The participant’s task was to respond “up” or “down” for the translational motion and “in” or “out” for the radial. Stimuli were displayed for a maximum of 16 s and were extinguished when a participant responded. A no response (failure to respond within the designated time) was considered to be incorrect. Stimulus trials were run using a 2 down-1 up staircase procedure with 8 reversals. Thresholds were calculated as the mean percentage of signal dots from the last 6 reversals. The staircase began at 100% coherence and had a proportional step size of 25% before the first reversal and 10% thereafter. The staircase was terminated prematurely if the participant gave incorrect responses for the first five trials, made two incorrect responses at the ceiling (100% coherence), or made two correct responses at the floor (2% coherence) of the staircase. If participants hit the ‘ceiling’ or ‘floor’ of the task prior to reaching the end of the criterion staircase (8 reversals), then they were scored according to the last recorded number of signal dots (i.e., 100% coherence (‘ceiling’) or 2% coherence (‘floor’). Mean response time was also recorded for each trial (time between stimulus onset and response).

Gaze position data were collected using an EyeTribe eye tracker (30 Hz, spatial resolution of 0.1°, accuracy of 0.5–1.0°)^25^ positioned below the display for trials involving simulated visual fields (i.e., excluding simulated acuity conditions). All raw gaze position data were reviewed for potential failings in the registration between eye position and the centre of the visual field prior to analysis. This check was essential in order to uphold the integrity of our simulated field conditions and avoid participants from seeing dot motion within an unintended area of their field (e.g., dot motion within the central field (<10°) during the >10° field condition).

### Visual acuity measurements

Static visual acuity was assessed using ETDRS visual acuity charts and the Berkeley Rudimentary Vision Test (BVRT) – White Field Projection (WFP) test card that can measure visual acuities to 2.9 logMAR^26^. The viewing distance was 4 m for the normal vision conditions and began at 1 m for the simulated visual acuity loss conditions. Charts were front-lit to a luminance of ~160 cd/m^2^ ± 10%^27^. Visual acuities were measured using a per-letter scoring system (0.02 units per letter)^28^ and participants were stopped once they reported ≥3 incorrect responses on a single line.

### Visual impairment simulations

Visual acuity loss was simulated using <0.1, 0.1, and 0.6 Bangerter foils^29^, which were combined with laminate sheets for the most severe visual acuity loss conditions. The Bangerter foils (and/or laminate sheets) were applied to plano lenses in Halberg clips that were placed on to participants’ habitual lenses or on to plano-lens spectacles supplied by the experimenter. Visual acuity impairments of ≥2.6, ≥1.5, ≥1.0, ≥0.4 logMAR were simulated. The first 3 conditions were synonymous with the Paralympic classification criteria for visual impairment (B1-B3 classes, Table 1) and the last condition was equal to the North American definition of low vision (≥0.4 logMAR; 20/50 or poorer)^30,31^.

**Figure 3:**
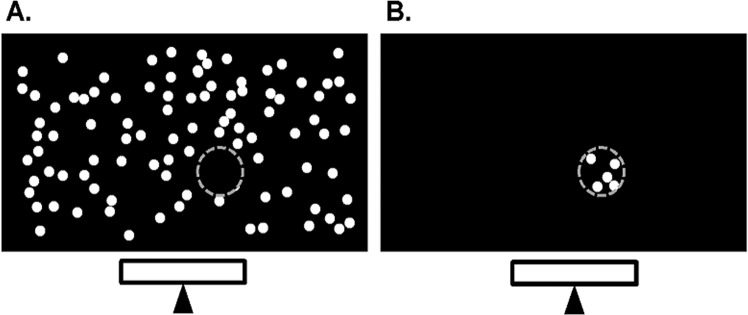
Illustration of the simulated visual field impairment conditions: peripheral (>10°) (**A**) and central (0–10°) (**B**). The lower rectangle represents the location of the gaze tracker. Dots were mapped with respect to a 10° diameter area, which was centred at the participant gaze location (as indicated by *grey dotted circle;* which was not present during the motion stimulus display). Note, images are not drawn to scale.

Two visual field loss conditions were simulated; a peripheral scotoma with a preserved central region of visual field and a central scotoma. In both cases, the central region of the visual field was circular with a diameter of 10°, which is consistent with the visual field criterion for the B2 Paralympic classification (Table 1). However, one Paralympic visual field criteria (B3, < 40° diameter) could not be tested due to display size limitations. During each trial, the position of the scotoma was updated in real-time based on the participant’s eye movements (Fig. 3). Therefore, the eyes were free to move around the display but the scotoma remained within the selected visual field area. The gaze trace was smoothed using EyeTribe’s custom proprietary filtering algorithm.

### Procedure

Participants attended three study visits. The first visit involved completion of the self-reported ocular health history form, a baseline measure of static acuity, determination of foil and laminate combinations for each of the simulated visual acuity impairments, and a baseline measurement of each motion coherence threshold (translational, radial) under normal viewing (no visual impairment). The second and third visits involved confirming the appropriate level of visual impairment by re-checking visual acuity with the selected foils and/or laminate and completing the global motion perception tasks under the simulated low vision conditions. In some cases, the filters needed a subtle adjustment at either visit 2 or visit 3. If an adjustment was made to participants’ filters, the adjusted filter was used for the remainder of the study. All reported simulated acuity thresholds are based on the final adjusted simulations.

Prior to starting the global motion task with the simulated vision impairments on the second visit, participants’ ability to detect the stimulus dots at the most severe visual acuity impairment condition (≥2.6 logMAR) was confirmed using a single dot detection task similar to the BVRT. A white dot (same size as the global motion task stimuli; 0.83°) was randomly presented in one of the four corners of the screen (8 presentations total), and participants were asked to indicate where they saw the dot.

Two consecutive staircases were run for each condition (normal vision and simulated impairment conditions) and the mean of the two thresholds was calculated to determine the coherence threshold. The order of stimulus motion (translation, radial) and simulated field impairments (central, peripheral) were counter-balanced across participants. The order of the simulated acuity impairments (≥2.6, ≥1.5, ≥1.0, ≥0.4) was randomized across participants. In total 28 global motion trials were completed across the three study visits (Visit 1 = 4 trials (normal vision; 2 translational and 2 radial), Visit 2 = 12 trials (three simulated impairments), Visit 3 = 12 trials (three simulated impairments).

### Data analysis

Three participants were excluded from the study due to initial problems with their gaze registration (final n = 15). Separate two-way repeated-measures Analysis of Variance (ANOVA) models were constructed: 2 motion (translational, radial) × 5 acuity (normal, ≥0.4, ≥1.0, ≥1.5, ≥2.6), and 2 motion (translational, radial) × 3 field (full, 0–10°, >10°). In the event of a violation of the assumption of equal variance of differences, as evaluated by Mauchly’s test, the Hynh-Feldt correction was applied when the Epsilon value was ≥0.75 and the Greenhouse-Geisser correction was applied otherwise (the original degrees-of-freedom are reported). Tukey HSD was used for post-hoc analysis and significance was declared at *p* < 0.05.

## Supplementary information

Appendix A.
