# Supplementary material for: Towards developing a test of global motion for use with Paralympic athletes
Source: Sci Rep. 2020 May 21;10:8482. doi: 10.1038/s41598-020-65202-x (PMC7242343; doi:10.1038/s41598-020-65202-x)
Supplement: Supplementary file 1 — Appendix A. [file 41598_2020_65202_MOESM1_ESM.docx]

**Towards developing a test of global motion for use with Paralympic athletes**

James W. Roberts^1, 2^, Benjamin Thompson^1^, Susan J. Leat^1^, & Kristine Dalton^1^*

**Appendix A. Supplementary Material**

**Method**

*Participants*

Five athletes with vision impairment who were participating in a national level classification event for vision impairment were recruited to undertake the global motion test (for participant characteristics, see Table A1).

Table A1. Participant characteristics

| Participant | Static Visual Acuity (logMAR) | Static Visual Acuity condition |
| --- | --- | --- |
| 1 | 1.38 | Tinted glass with no Rx |
| 2 | 1.6 | Unaided |
| 3 | 2.0 | Unaided |
| 4 | 0.26 | Aided |
| 5 | 0.64 | Aided |

*Stimuli and Procedure*

The same dot motion parameters as the simulated low vision study were adopted for the testing of athletes with the exception that only the translational motion stimuli were displayed and the dots were sized at 0.17° (2.91 mm) or 1.67° (29.16 mm) (equating to a single limb width of a 1.0 and 2.0 logMAR optotype, respectively).

Prior to undertaking the global motion test, participants were first required to correctly discriminate at least 6 out 8 displays of a 100% coherent motion display by responding to the “up” and “down” keys on the response pad. In the event they were unable to reach this criterion, it was assumed that they would be unable to undertake the global motion test, and consequently allocated a maximum coherence threshold (100%). Participants completed one trial for each of the dot sizes. The dot coherence was modulated by a 2 down-1 up staircase procedure until there were 6 reversals. Thresholds were calculated as the mean percentage of the last 4 reversals.

**Results**

The data were analysed by averaging the coherence thresholds across both dot sizes for each of the participants. The data are plotted as a function of the athletes’ static visual acuity in Figure A1. Similar to our findings with simulated low vision, it appears possible for global motion perception to occur with significant reductions in visual acuity (2.0 logMAR visual acuity). However some participants with better visual acuity (1.38 and 1.6 logMAR) did very poorly on this task. That said, there was a general decline in global motion perception with reduction in in acuity, which may be attributed to a reduced ability to detect the local motion signals (V1) that precede global integration (MT/V5) (Morrone, Burr, & Vaina, 1995).

Figure A1. Participant’s mean coherence thresholds (%)

as a function of static visual acuity (logMAR)
